# Current respiratory support practices in premature infants: an observational study

**DOI:** 10.11604/pamj.2021.39.66.14482

**Published:** 2021-05-25

**Authors:** Kaoutar Khabbache, Yves Hennequin, Daniel Vermeylen, Bart Van Overmeire

**Affiliations:** 1 Neonatal Intensive Care, Hôpital Erasme, Université Libre de Bruxelles, Bruxelles, Belgique

**Keywords:** Invasive ventilation, non-invasive ventilation, premature infants, ventilatory support

## Abstract

This study aims to describe longitudinally the current invasive and non-invasive ventilation practices in premature infants in a single neonatal intensive care unit (NICU). It´s a retrospective chart review including 682 babies born at gestational age ≤35 weeks, admitted to the NICU at Erasme Hospital, between 1^st^ of January 2001 and 31^st^ of December 2011, the different ventilatory support used were analyzed. This population was stratified depending on gestational age and the recruitment period on 3 groups. All infants born <28 weeks of GA (group 1) needed some kind of respiratory support of which 22% non-invasive. Among babies born after 28 to 31 weeks (group 2), 10.2% didn´t need any ventilatory support and 42% needed a non-invasive respiratory support. In neonates from 32 to 35 weeks of GA (group 3) respiratory support was needed in 34.9%, 65% of which was non-invasive. The median duration of endotracheal ventilation was: 6, 1 and 2 days, and of non-invasive support: 41, 17 and 2 days in group 1, 2 and 3 respectively. One single premature baby could pass along the first weeks through all modes. In premature infants whose respiratory support was needed, the median age at the end of support was remarkably constant at 33 - 34 weeks of corrected age. We conclude that is an important diversity and a significant complementarity between modes of respiratory support for premature infants. Invasive ventilation decreased significantly for group 2, but is still remarkably long for group 1.

## Introduction

A large portion of premature infants present respiratory failure after birth because of reduced functional residual capacity due to surfactant deficiency, immaturity of the respiratory tract and evolving forms of chronic lung disease [1,2]. This respiratory distress often presents diagnostic and management challenges to the attending neonatologists. It is known that early intervention in babies with acute respiratory distress often prevents further complications. The main goals of management of respiratory failure in the premature infants are to maintain vital functions, minimize iatrogenic injury and optimize long term outcomes [3]. Currently there is an important progress in neonatal respiratory care. Premature infants require different techniques of invasive and non-invasive mechanical ventilation. Unfortunately, the advancement of the technology has occurred over a very relatively short period of time, limiting the ability to establish an evidence-based approach for comparisons and decision-making [4]. However, this should not preclude clinicians from using the principles of pulmonary mechanics and respiratory physiology in applying these techniques to ill newborns.

In our practice the different types of invasive ventilation modes used for premature infants are: synchronized intermittent Conventional Mechanical Ventilation (CMV), High frequency oscillation (HFO), and non-invasive or “less invasive” ventilation modes including Nasal Continuous Positive Airways Pressure (NCPAP), non-Invasive Intermittent Positive Pressure Ventilation (nIPPV) or Bilevel nasal CPAP (BIPAP), High Flow Nasal Cannula (HFNC) and Low Flow Nasal Cannula (LFNC). The principles and application of these techniques have largely been reported [5,6], but few studies have described practically the proportion of use, the integration and experience of each technique in an individual NICU (Neonatal Intensive Care Unit). In particular our data identify the duration of the respiratory support by category of gestational age, demonstrate the diversity of techniques used and emphasize the attempts to limit the duration of invasive ventilation associated with surfactant administration. We describe the different ventilatory modes and how they are applied in a tertiary neonatal care center in Belgium with focus on the evolution of invasive ventilation in the last decade, a period during which the InSurE technique (intubate, surfactant administration, extubate) has been progressively introduced.

**Aim:** to describe modern ventilation practices in premature infants in a single level 3 NICU; to show the diversity of different ventilatory modes, the proportion and the duration of their use and the transition between different modes according to gestational age; to evaluate the impact of a non-invasive ventilation approach and InSurE in reducing duration of invasive ventilation.

## Methods

**Patients:** a retrospective chart review was conducted on 330 babies born at gestational age ≤35 weeks, who were admitted to the NICU at Erasme Hospital, between 1^st^ of January 2010 and 31^st^ of December 2011. Neonates who were transferred to other hospitals before 24 hours of life (n=2) were excluded. Additional data was collected from infants with gestational age <32 weeks admitted between January 1^st^ 2001 and 31^st^ of December 2011 and who needed endotracheal intubation.

**Description of the different ventilatory supports used:** HFO: High Frequency Oscillation (SLE 5000 infant ventilator) is used in our practice on first intention in premature infants with respiratory distress at birth who need an invasive ventilation. CMV: Conventional Mechanical Ventilation (Babylog 8000): synchronized ventilation with targeted volume is used most of the time as a secondary ventilatory invasive support after HFO. BIPAP: Bilevel Nasal Cpap or Non-invasive intermittent positive pressure ventilation (nIPPV) (Infant flow SiPAP), is used in none synchronized biphasic mode, with Peep (positive end expiratory pressure) at 3-5cm H_2_O and PIP (Positive Inspiratory Pressure) at 5-7cm H_2_O. NCPAP: Nasal Continuous Positive Air Pressure (Infant flow TM Nasal CPAP) with Peep between 3-5 cm H20. Nasal prongs or mask are used depending on what is best tolerated by the infant. HFNC: Humidified and Warmed High Flow Nasal Cannula (Fisher & Paykel) are used at 1L/min/Kg + 1L/min with cannula´s diameter less than half the nostrils. LFNC: Low Flow Nasal Cannula are used with flow less than <1L/min. Surfactant is used as early or late rescue therapy. Indications and weaning from different ventilatory modes and the technique of InSurE used in our unit are based on recent literature [7]. Statistical analysis was done by software graph pad prism 6 using nonparametric Mann-Whitney test.

## Results

**Figure 1:**
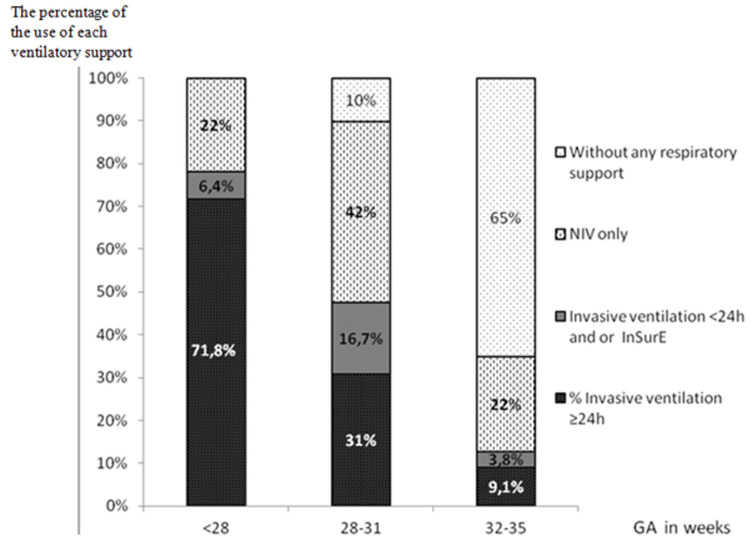
repartition of the need and invasivity of ventilatory support according to gestational age (NIV: non-invasive ventilation, InSurE: intubate-surfacte-extubate, GA: gestational age)

A total of all 330 premature infants´ ≤35 weeks of GA were identified during the recruitment period (1^st^ of January 2010 and 31^st^ of December 2011) and stratified by GA as follows: (Group 1): <28 weeks of GA (n = 32). (Group 2): between 28 and 31 of GA (n=88). (Group 3): between 32 and 35 GA (n=210). There were 4 deaths in group 1, 2 deaths in group 2 and 2 deaths in group 3. It was noticed that all premature infants <28 weeks of GA (group 1) needed respiratory support, however not all of them required intubation and surfactant, 22% of them were managed only with non-invasive support. In the group 2: 10% didn´t need any ventilatory support and 58.7% of cases needed a non-invasive support or an invasive one but less than 24 hours. In the last category group 3, premature babies needed a respiratory support in 34.9%, and only non-invasive support in 22% (Figure 1). Concerning the technique of InSurE it was used in 6.25% cases for group 1, 13.63% for group 2 and 2.85% for group 3. Short duration of intubation or InSurE succeeded in 8% of intubated premature babies in Group 1, 35% in group 2, 70.54% in group 3.

**Figure 2:**
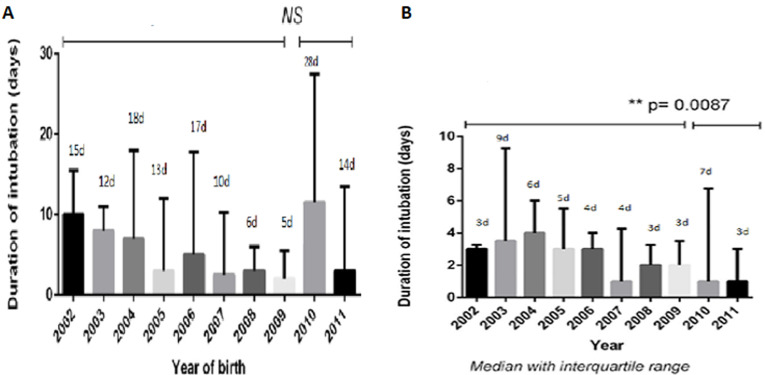
A) duration of intubation in the last decade for group 1 (less than 28 weeks of GA); (NS: not significant); B) duration of intubation in the last decade for group 2 (28-31 GA weeks)

The median duration of endotracheal ventilation according to GA is 6 days (IQR: 1-18.5) for premature <28 weeks of GA, 1 days (IQR: 0-3) for premature between 28 and 31 weeks of GA and 2 days (IQR: 0-3.5) for premature between 32 and 35 GA. In the complementary study group of premature infants <32 weeks with endotracheal intubation (1^st^ January 2010 and 31^st^ of December 2011), we observed a significant decrease of duration of intubation from 2009 onward in group 2 (p=0.0087) but not for group 1 (Figure 2 A,B). This was related to the introduction of InSurE in the unit during the same period. The median duration of non-invasive ventilation is obviously depending on GA. It´s particularly long in the premature babies Group 1: 40.5 days (IQR: 28-56.25). For group 2 it's 17 days (IQR: 7.5-30), and it´s just 2 days (IQR: 1-6) for group 3. Currently in our practice we use different ventilatory modes for all categories of premature infants to manage their respiratory failure, as well as invasive or non-invasive mode. In premature <28 weeks of GA BIPAP is the main mode used.

**Figure 3:**
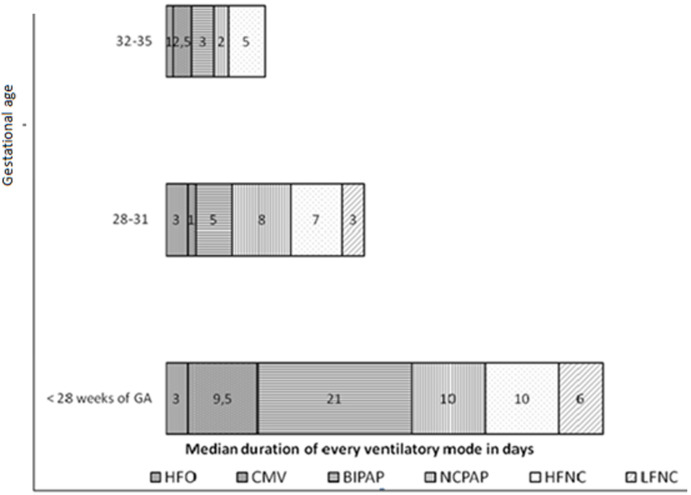
median duration of each ventilatory mode according to GA (HFO: high frequency oscillation, CMV: conventional mechanical ventilation, BIPAP: Bilevel nasal Cpap, NCPAP: nasal continuous positive air pressure, HFNC: high flow nasal cannula (humidified and warmed), LFNC: low flow nasal cannula)

**Figure 4:**
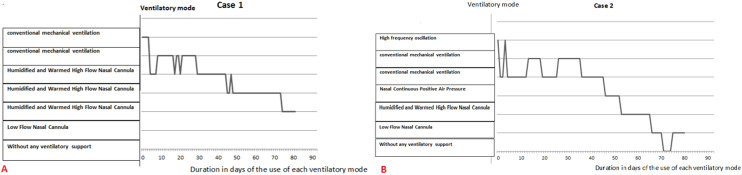
A) example of transition from a ventilatory mode to another one in case 1 (premature of 25 GA); B) example of transition from a ventilatory mode to another one in case 2 (premature of 26 GA)

For 28-31 weeks of GA NCPAP is the most used. In premature infants between 32 and 35 weeks of GA HFNC is the mode that is using the longest time (Figure 3). The median of corrected age at weaning of any ventilatory support is for group 1; 34 weeks with a maximum at 41 weeks, for Group 2 it´s 33 with maximum at 37 weeks, for Group 3 it´s 34 with a maximum at 37 weeks. Indeed when premature infants need a respiratory support the median of the end of support is remarkably constant or similar at the corrected age of 33 - 34 weeks. The diversity of ventilatory modes used is illustrated by the cases of 2 very premature babies: case 1 born at 26 weeks 3 days (Figure 4 A), and case 2 born at 25 weeks 5 days (Figure 4 B). These premature babies passed progressively through all modes of ventilation. The weaning from an invasive ventilatory support to another less invasive was not always successful with return to a more invasive one. Four intubations were needed for the two cases with a very long total duration of ventilatory support; it reached 81 days for case 1 and 80 days for case 2.

## Discussion

Over time, technological advances in ventilators have provided neonatologists with new ventilation modes and techniques, many of which have been tested in randomized controlled trials [8]. However, the translation and implementation of these trials into daily clinical practice has been poorly studied. This paper describes how neonatal ventilation in premature infants is practiced in a NICU. Although the trend in the NICU is to prefer non-invasive ventilation whenever possible and to limit the duration of invasive ventilation with endotracheal tube as short as possible, this last is still necessary especially for extremely preterm neonates. As expected, in our data all premature infants less than 28 weeks of GA needed ventilatory support, most of them invasive ventilation more than 24 hours. Only 16% in this group will never be intubated or receive surfactant. A recent survey of 173 European neonatal units highlighted that in a predominately prematurely born population (mean gestational age 28 weeks and birth weight 1024 g); the median duration of mechanical ventilation was 4 days (IQR 1-12) (interquartile range) [9]. In our study with a population exclusively below 28 weeks, the median was 6 days (IQR: 1-18.5). In the same survey, 85% of patients were conventionally ventilated versus 15% in HFO mode. It shows that TCPL (Time cycled pressure-limited ventilation) combined with a synchronized intermittent mandatory ventilation (SIMV) synchronization mode, is the most popular conventional ventilation mode for ventilating both preterm and term newborn infants [9]. Whereas in our NICU, HFO mode is used as a primary invasive mode for premature infants and if necessary, passing then to a conventional ventilation with volume guarantee. There is an ongoing debate whether neonates who need invasive ventilation at birth, should be primarily managed by HFO or CMV [10].

A promising new form of patient-triggered ventilation, neurally adjusted ventilatory assist (NAVA), was recently FDA (food and drug administration) approved for invasive and non-invasive ventilation. Clinical trials are underway to evaluate outcomes in neonates who receive NAVA [11]. But we don´t have any experience yet in our NICU. In group 2, InSurE strategy was more successful which leads to a shorter duration of invasive ventilation (1 day). In group 3, this was slightly longer (2 days) due to post surgery invasive support (5 cases; 18.5%). In our data, NCPAP is the mode the longest used for group 2. In the literature, NCPAP seems the most frequently used in preterm infants [12]. It has been shown that NCPAP may reduce the need for invasive intubation and ventilation; reduce apnea of prematurity and post extubation atelectasis. Early use of NCPAP reduces the incidence of bronchopulmonary dysplasia and the need for home oxygen [13,14]. However, NCPAP therapy is associated with complications as nasal trauma, gastric distension, obstruction by secretions requiring more labor by health care team, and overall perceived patient discomfort and there is very little evidence on the best methods of weaning babies off NCPAP [15-17]. The longest and the most frequently used ventilatory mode in premature <28 weeks in our practice is BIPAP. It´s reported that BIPAP may improve ventilation by maintaining patency of the upper airway and by promoting triggering of respiratory reflexes [17]. Smaller studies demonstrate that BIPAP may be an alternative to intubation in premature babies who have deteriorated despite standard NCPAP therapy [18]. There is a suggestion of reduced bronchodysplasies in extremely preterm infants treated with BIPAP, but there are as yet no randomised trials to support this suggestion [19]. In our data bronchopulmonary dysplasia affected 32% of premature < 31 weeks of GA (68.75% of premature group 1, and 18.60% of premature group 2). In other reports it affects approximately 20-40% of very-low-birth weight [20].

In our practice and in the literature, high flow nasal cannula is now being used more frequently for both weaning and as a replacement of NCPAP and BIPAP [21]. Humidified and Warmed High Flow Nasal Cannula is preferred by caregivers due to its ease of administration and the ability to feed and care for the infant while on this mode of ventilatoty support. Infants cared with HFNC can easily interact with parents and environment which could be developmentally advantageous [22]. Some recent data suggest the use of HFNC for primary respiratory support at higher flow of 4-8 L/min. Furthermore, this high flow warrants additional precautions [22] and more research is needed with these alternative less invasive forms of respiratory support [23]. When ventilatory support was needed, the total duration in our patients remained remarkably constant up to a median of 33 - 34 weeks of corrected age for the 3 groups. This duration is still long for the most premature babies despite progress in ventilation. There is unfortunately no published data available describing the total duration of any respiratory support. The most papers focus on one mode or on one category of GA. Our study includes all categories of premature GA and all ventilatory modes, so it´s difficult to compare our data. The duration of endotracheal ventilation significantly decreased since 2009 in group 2, it´s related to the introduction of InSurE in our practice in this period. It´s recognized that babies receiving InSurE have less need for mechanical ventilation, fewer pneumothoraces and less bronchopulmonary dysplasia [24], but evidence of long-term benefit is limited [25]. Since moderately preterm babies are likely to have a greater respiratory drive and a more effective respiratory effort than extremely preterm infants, the InSurE technique may offer the opportunity to administer surfactant at the first sign of respiratory distress syndrome and then successfully extubate to NCPAP. In our practice and at this time, InSurE is not yet an easy and current practice for the most premature infants of less than 28 weeks.

One single premature baby can pass through all modes progressively from one technique to a less invasive one. Transition and weaning are not always successful with a risk of repeated intubations for the most premature babies. The variability and diversity of the ventilatory modes which we have currently offer us a wide choice. Certainly, all modes are complementary and indispensable for our practice included the newest one. But this multiplicity could create confusion if it is not accompanied by protocols, training and monitoring of its effects and risks.

## Conclusion

We demonstrated an important variability, diversity and complementarity between invasive and non-invasive modes of respiratory support for premature infants. The objective is to promote non-invasive ventilation and to diminish the total duration of ventilatory support. But it is still a challenge particularly for the most premature ones who need long periods of invasive and non-invasive support. When used properly, these complementary modes may have advantages in terms of reducing neonatal lung injury and improving outcomes in premature babies requiring mechanical ventilation. The duration of each mode and the total duration of the support according to gestational age are decreased mainly for the 28-32 weeks but are still long for those below 28 weeks. This is due to the introduction of InSurE and the amelioration of non-invasive support. These data on ventilation practices in premature infants including all respiratory support modes and the changing of different modes emphasize that further research is required to compare ventilation modalities or strategies and to produce evidence-based guidelines for the respiratory support of premature infants.

### What is known about this topic

- The principles and indications of each ventilatory support have largely been reported;
- Most current research evidence relates to the weaning of one mode of respiratory support to another less invasive one;
- The interest of InSurE in short intubation is already recognized.

### What this study adds

- Description of how the different ventilatory modes are applied in a NICU and illustrate the difficulty of management of respiratory failure in very premature infants;
- To note the total duration of the respiratory support, the proportion of use and the integration of each technique by category of gestational age;
- To show the evolution of the duration of invasive ventilation in the last decade.
